# Waste Plastic Polypropylene Activated Jujube Charcoal for Preparing High-Performance Phase Change Energy Storage Materials

**DOI:** 10.3390/nano13030552

**Published:** 2023-01-29

**Authors:** Xifeng Lv, Huan Cao, Rui Zhang, Xuehua Shen, Xiaodong Wang, Fang Wang

**Affiliations:** 1 The National and Local Joint Engineering Laboratory of High Efficiency and Superior-Quality Cultivation and Fruit Deep Processing Technology of Characteristic Fruit Trees in South Xinjiang, College of Chemistry and Chemical Engineering, Tarim University, Alar 843300, China; 2 State Key Laboratory of Organic-Inorganic Composites, Beijing 100029, China; 3 School of Environmental Engineering and Chemistry, Luoyang Institute of Science and Technology, Luoyang 471023, China

**Keywords:** polypropylene, jujube charcoal, polyethylene glycol, phase change materials

## Abstract

The research on the high-value utilization of biomass has good application prospects and is conducive to sustainable development. In this paper, three different types of activators (potassium hydroxide, phosphoric acid, and polypropylene) were used to carbonize jujube branches at high temperatures of 600 °C and 800 °C, and then the PEG/jujube charcoal composite phase change materials (PCM) were prepared by vacuum impregnation of polyethylene glycol (PEG). The results showed that the carbon support activated by polypropylene (PP) had a richer pore size distribution than the other two activation methods, and the 800 °C carbonization carrier loaded PEG had a higher phase change enthalpy than the composite material at 600 °C. The mesoporous and macroporous structures were staggered with PP-activated jujube charcoal at 800 °C, with a specific surface area of 1082.2 m²/g, the melting enthalpy of the composite material reached 114.92 J/g, and the enthalpy of solidification reached 106.15 J/g after PEG loading. The diffraction peak of the composite phase change material was the superposition of PEG and carbon matrix, which proved that the loading process was physical adsorption. After 200 thermal cycles, the melting enthalpy and crystallization enthalpy were only reduced by 4.3% and 4.1%, respectively, and they remained stable and leak-free at the melting point of PEG for 2 h, demonstrating good thermal stability of the composite phase change materials. In summary, PP has obvious advantages over traditional activation, and the carbon-supported PEG phase change composite after PP activation is a biochar energy storage material with excellent performance.

## 1. Introduction

The energy crisis has led to an increasing interest in new energy materials represented by phase change materials. Research has found that thermal energy storage (TES) using phase change materials (PCM) is suitable for space heating [1] and has the ability to reduce the energy demand of buildings [2]. Xinjiang is very suitable for the cultivation of red dates due to its special climate. According to data from the China Bureau of Statistics, in 2020, Xinjiang’s red date planting area and output ranked first in the country. At present, the planting area exceeds 6 million mu, accounting for nearly one-third of the total planting area in the country, and the planting area has shown an increasing trend in recent years. The pruning of jujube trees is extremely important to increase the yield of red dates. Spring date tree pruning is the top priority of the year’s production task, and the branches pruned by date farmers can only be stacked on the roadside and in ditches, which not only affects traffic and the village appearance, but also has certain safety risks. At the same time, the increase in the production of plastic waste worldwide has become an urgent issue of our time. Among them, polypropylene (PP), as a typical non-biodegradable plastic, will threaten the natural ecological environment for a long time. Turning waste into treasure has become an effective way to solve plastic waste. With its high compressive strength and excellent porous structure, jujube branches are very suitable for the preparation of highly stable biochar materials.

There have been some reports to study the pore structure control method of porous carbon materials. For example, Zhang et al. [3] showed that the system control of the pore structure is conducive to its efficient performance and high-density energy storage. Additionally, the proportions and activation methods of different raw materials, components, and activators have a great influence on the quality and application of carbon materials [4]. At present, the methods of pore structure activation mainly include physical activation (water vapor, carbon dioxide) and chemical activation (phosphoric acid, sulfuric acid, potassium hydroxide). Yin’s group [5] proved that compared with chemical activation, physical activation usually has a lower yield, low porosity, small pore size, high activation temperature, and long activation time. Özer et al. [6] prepared a new biochar from pumpkin peel by phosphoric acid activation. The results showed that the new biochar had a higher specific surface area and micropore structure, which were 689.9 m^2^/g and 6.0 Å, respectively. He and his coworkers [7] studied KOH activated porous carbon and found that the hierarchical porous biochar extracted from the KOH activated Soulangeana sepals had a hierarchical porous structure, high specific surface area, and abundant oxygen-containing functional groups. Jie et al. [8] showed that the thermal decomposition of plastic, as a common waste, and a representive hydrogen-rich energy raw material with high hydrogen content (8%~14%) could produce hydrogen-rich gas. Studies by Jia et al. [9] and Luo et al. [10] showed that gases (CO, CO_2_) are essential for pore formation. Therefore, it is foreseeable that PP can promote the formation of pores during the pyrolysis process of biomass. Ma’s group [11] demonstrated that certain plastic products such as PP could improve the stability of the biomass. In addition, Tang et al. [12] showed that PP with a certain small particle size could accelerate the pyrolysis of biomass. Several studies by Xu et al. [13] showed that the co-pyrolysis of biomass with hydrogen-rich raw materials such as plastics is an effective way to reduce coke formation. The research of Li et al. [14] also showed that PP is considered to be an excellent co-pyrolysis additive, which can be used as a supplement to hydrogen for promoting the formation of pores and generating porous carbon materials.

The phase change material is mixed with the building material to prepare a new type of high-efficiency and energy-saving phase change energy storage material, which can well control the temperature change of the building and improve the temperature resistance of the building. Shape-stabilized phase change composites are the main research direction and have important application value. At present, there are three main methods for preparing and forming composite phase change materials, the microcapsule method, porous material adsorption method, and melt blending method. Su’s group [15] reviewed the energy storage applications of various types of solid–liquid phase change materials and summarized several better methods for preparing and forming composite phase change materials. Darkwa et al. [16] developed a non-deformed phase change energy storage material with high thermal conductivity and high energy storage density through the compaction process, which can effectively reduce the porosity of the composite and improve the conductivity and energy storage density. At the same time, encapsulating phase change materials with porous activated carbon as an adsorbent is also an effective method for preparing phase change energy storage materials. For insistence, Mesalhy et al. [17] prepared phase change energy storage composites by using carbon foam and paraffin with different pore structures. The results showed that the higher the porosity of carbon foam facilitates the greater the adsorption capacity of paraffin, achieving a higher latent heat of the composite. The application of phase change materials provides an effective approach for the development of new energy sources [18].

Polyethylene glycol (PEG), as a phase change material, has high latent heat and high cost-performance. Wang et al. [19] used PEG as a heat storage material to prepare porous carbon-based phase change materials by simple blending impregnation, and the enthalpy value of PEG in the composites could be extremely well-controlled. Shen’s group [20] studied and prepared a biomass-based carbon aerogel as a porous support material, and magnetic Fe_3_O_4_@polyethylene glycol was prepared by the vacuum impregnation method. Biomass-based carbon aerogel (LG)-based phase change composites with a stable shape were obtained, which have good thermal storage properties, high latent enthalpy, and excellent electrical conductivity. In this work, a composite phase change energy storage material with good heat storage effect was prepared by vacuum impregnation using jujube activated carbon as the adsorption carrier and PEG as the phase change material. The activation of biomass activated carbon materials is traditionally based on acid and alkali, which is not friendly to environmental protection, and our work used PP as the raw material for biochar pore formation, aiming to prepare a new phase change energy storage material with energy saving, environmental protection, and high efficiency. The material is suitable for low temperature space heating and has the ability to reduce the energy demand of buildings. Therefore, it has broad application prospects in building heat storage [21].

## 2. Materials and Methods

### 2.1. Materials

Potassium hydroxide (KOH) was purchased from the Tianjin Damao Chemical Reagent Factory, China. Hydrochloric acid (HCl) was provided by Sichuan Xilong Chemical Co. Ltd. of China. and phosphoric acid (H_3_PO_4_) was ordered by Tianjin Beilian Fine Co. Ltd. of China. Polypropylene was bought from Tianjin Bodi Chemical Co. Ltd. of China, Polyethylene glycol-800 (8P) was offered by Xinjiang Lanshan Tunhe Polyester Co. Ltd. of China and potassium bromide (KBr) was purchased from Shanghai Xinghuo Chemical Plant of China.

#### 2.1.1. Preparation of Porous Jujube Charcoal

Pretreatment of jujube branches. The jujube branches were placed in a blast drying box at 80 °C for 6 h, before a knife was used to cut the bark off the jujube branches, then the jujube branches were cut with scissors, and then were dried in an oven at 80 °C to constant weight. After drying, the jujube branches were removed from the drying oven and allowed to cool. These were then put into the crusher in batches many times, and after passing a 100-mesh standard sieve, the material was put in a dry place for storage.

KOH-activated jujube charcoal. Two times 1.5 g jujube sprig powder were first added into a beaker containing 40 mL of distilled water with KOH in a ratio of 1:3. The solution was stirred thoroughly under a magnetically heated stirrer for 2 h and dried to constant weight. Two parts of the treated jujube powder were continuously heated in a tube furnace at 600 °C and 800 °C, respectively, for 1 h, and nitrogen was used as the protective gas and the heating rate was 4 °C/min. Heating was stopped after activation, but nitrogen was continued to cool it down. After cooling, it was put in two beakers containing distilled water and sonicated for 30 min. The pH was adjusted to neutral with the configured 0.1 mol/L hydrochloric acid solution. It was washed repeatedly with hot distilled water, and finally dried at 60 °C for later use, named 6CK and 8CK, respectively.

H3PO4 activated jujube charcoal. Two parts of 1.5 g jujube branch powder were put in a beaker with phosphoric acid at a ratio of 1:2 and impregnated with a seal for 24 h. After that, the solution was poured into a porcelain crucible and formed charcoal at 600 °C and 800 °C, respectively, in a tube furnace, and the charcoal formation environment was the same as for KOH-activation. After cooling, it was put into distilled water and sonicated for 30 min. Then, it was washed repeatedly with hot distilled water until neutral, and finally dried at 60 °C for reserve, named 6CH and 8CH, respectively.

PP activated jujube charcoal. Two parts of 1.5 g jujube branch powder were weighed and mixed well with PP (a mass fraction of 30%) in a twin-cone screw extruder. After cooling, it was poured into a porcelain crucible and placed in a tube furnace to form charcoal at 600 °C and 800 °C, and the charcoal formation environment was the same as KOH-activation. After cooling, it was sonicated for 30 min, repeatedly washed with 0.1 mol/L hydrochloric acid solution and hot distilled water to neutral, and finally dried at 60 °C for reserve, named 6CP and 8CP, respectively.

#### 2.1.2. Preparation of Composite Phase Change Materials

Taking 6CK, 6C,P and 6CH as the composite material carriers, 50%, 60%, 70%, and 80% of PEG were added, respectively, and then put into a vacuum drying oven. After 4 h of immersion at 30 °C and 0.1 MPa, the optimal loading amount of PEG was determined by the experiments. After that, 80% PEG was added in 6CK, 6CP, 6CH, 8CK, 8CP, and 8CH, and impregnated at 30 °C and 0.1 MPa for 4 h. The filter paper was used to suck out excess PEG repeatedly. The final composite phase change material was named 6CPK, 6CPP, 6CPH, 8CPK, 8CPP, and 8CPH.

### 2.2. Characterization

X-ray diffraction (XRD) was performed with a D8 Advance (Bruker, GER). Scanning electron microscopy (SEM) images were conducted on a JSM-7800F (Jeol, Japan). X-ray photoelectron spectroscopy (XPS) was implemented using Thermo Scientific K-Alpha (China). The specific surface area and pore size distribution of the samples were obtained from their N_2_ adsorption–desorption curves, obtained using a Micrometric ASAP 2020 instrument (USA). The infrared thermography test was finished with a HM-TPH21 Pro-3AQF thermal imaging camera (HIKMICRO, China). DRL-III thermal conductivity tester (Xiangtan, China)was used to detect the thermal conductivity of composite phase change materials and the Nicolet iS10 infrared spectrometer was applied to characterize the Fourier transform infrared spectroscopy (FTIR) of the composite phase change materials (Thermo Fisher Scientific instrument, USA). Phase change behaviors of the PCMs were studied by differential scanning calorimetry (DSC, DSC-214, Netzsch, Germany) at a heating/ cooling rate of 10 °C/min in N_2_. 

## 3. Results

**Figure 1:**
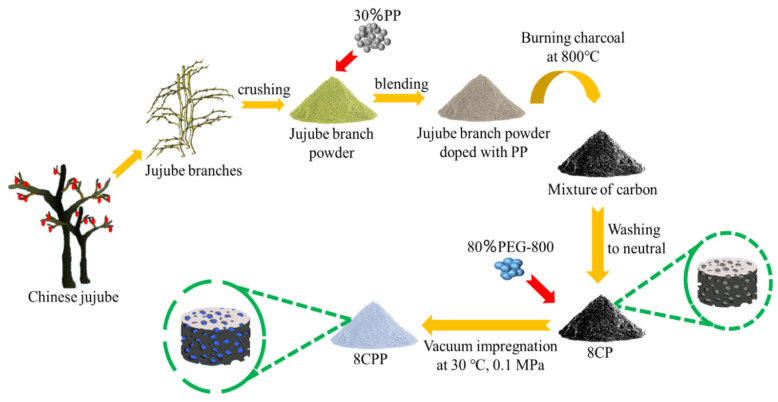
The preparation process for the 8CPP.

**Figure 2:**
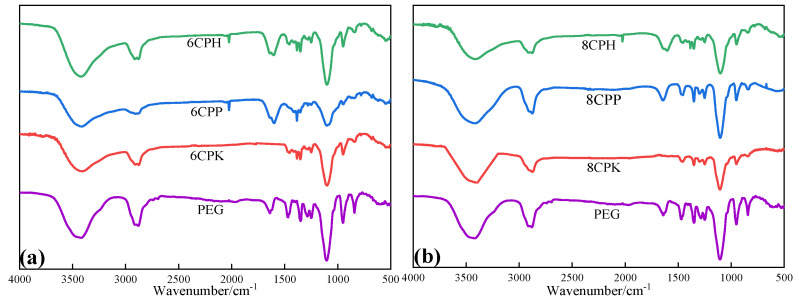
Infrared spectra of the composites and pure PEG under different temperature activation. (**a**) FTIR of PEG and the composites after activation at 600 °C; (**b**) FTIR of PEG and the composites after activation at 800 °C.

Figure 1 depicts the schematic diagram of carbonization of waste jujube branches under the action of PP to prepare mixed biochar and the phase change materials by PEG loading. The gas released during the pyrolysis of PP can promote the formation of pores in the carbonization process of jujube branches and generate porous biomass carriers. In a vacuum environment, the capillary force generated by this porosity has a strong adsorption effect on PEG, which is expected to have a significant effect on the leakage prevention of the phase change material. Infrared spectroscopy research was mainly used to study the surface groups of the composite phase change materials prepared. As shown in Figure 2, the absorption peak at 3435 cm^−1^ was caused by the telescopic vibration of –OH; the absorption peak at 2890 cm^−1^ was caused by the telescopic vibration of –CH; the absorption peak at 1630 cm^−1^ attributed to C=O; and the absorption peaks at 2890 cm^−1^, 1460 cm^−1^, and 945 cm^−1^ together constitute the characteristic peaks of the –CH group [22]. The location of 945 cm^−1^ was also the PEG crystal peak, 1245 cm^−1^ was the absorption peak of the OH bending vibration, the absorption peak at 1110 cm^−1^ was the telescopic vibration of C–O–C, and the absorption peak at 840 cm^−1^ was caused by the C–C–O bond [23]. Porous carbon made under the condition of inert gas protection cannot avoid combining with oxygen in the air to form oxygen-containing groups such as C=O, –OH, and –C–O, which will affect the surface structure of the composite. Overall, the infrared absorption peak of the composite phase change material contained the characteristic absorption peak of PEG, and no new structural characteristic peak appeared, indicating that there was no chemical reaction between PEG and jujube charcoal during the composite process, and the carbon material and PEG were composed of only physical effects [24]. The wavenumber change corresponding to some groups of PEG and the composite materials indicates that there may be some force between PEG and jujube charcoal. Since both PEG and the composites have OH, it is speculated that there is hydrogen bonding between PEG and the carbon matrix of jujube branches, which restricts the leakage of PEG in the molten state from the carbon matrix of the jujube branches. Comparing the peak positions of each group in Figure 2a,b, it was found that there was no significant difference between the two temperatures, and only some of the peak intensity increased or decreased.

**Figure 3:**
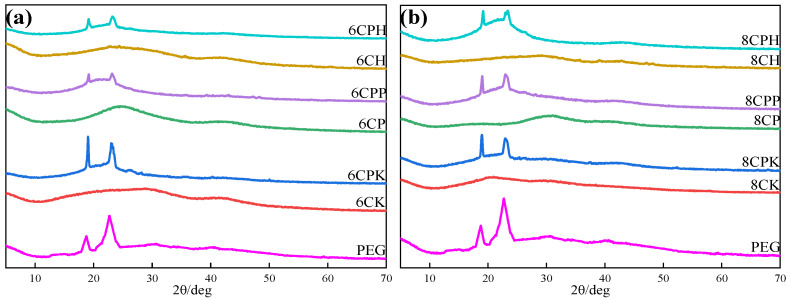
XRD spectra of PEG, carbon materials, and composites under different temperature activation. (**a**) 600 °C, (**b**) 800 °C.

XRD characterization was used to further determine the composition and microstructural changes of the composite phase change materials. As shown in Figure 3, the diffraction peak position of PEG in the composite material did not change compared to pure PEG, indicating that porous materials do not affect the crystal structure of PEG [25]. This is consistent with the FTIR results, where no chemical reaction occurred between PEG and the porous material. However, it can be clearly seen that the PEG peaks in the composite were different from the pure PEG peaks because the interactions between the carbon materials and between the carbon materials and PEG such as capillary forces and hydrogen bonds can hinder the movement of the polyethylene glycol segment during the crystallization process. It can be seen from Figure 3a that the peak value of 6CPK corresponding to 6CK after KOH activation was higher, which was due to the fluffy and soft texture of the carbon material prepared by KOH activation, and the force between it and PEG was extremely small, which hardly limited the crystallization process of PEG, and the other two activations showed relatively large forces. The peak value was not high, but observing the peak area of each composite material, it could be seen that the amount of PEG loaded was still large, reflecting a good loading effect. Observing the peak situation of Figure 3b, it can be understood that the peak height of the three materials was approximately the same, but the peak area was obviously different, and the peak area of 8CPP and 8CPH was higher, indicating that the amount of PEG loaded was greater, while the peak area of 8CPK was relatively low.

**Figure 4:**
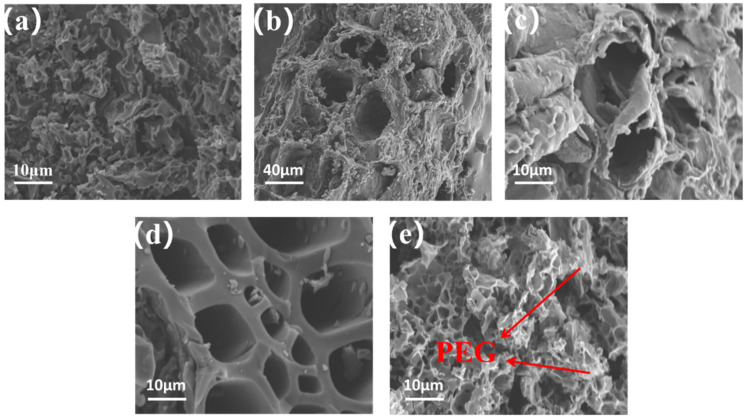
(**a**–**c**) SEM pictures of 8CK, 8CP, and 8CH; (**d**) a sectional SEM diagram of 8CP; (**e**) SEM photos of 8CPK.

The surface topography of the prepared composite was observed by scanning electron microscopy, and the test results are shown in Figure 4. As can be seen from Figure 4a, the surface of 8CK was full of micropores, and its matrix structure was mostly superimposed in sheets. Although such a structure can load a considerable amount of phase change materials, it has a long loading time due to its small and many pores layered on top of each other. From the surface of 8CP shown in Figure 4b, it can be seen that it had an elliptical three-dimensional structure, with staggered distribution of macropores and mesopores. Generally, macropores play a role in unblocking when PEG is loaded, and mesopores play a key encapsulation role when PEG leaks, and each performs its own function to ensure the excellent performance of the composite phase change material prepared by the carbon matrix. The same conclusion has been obtained in previous reports [26]. Figure 4c shows the SEM diagram of 8CH, which had a fluffy three-dimensional shape, large pores, and a slightly smaller number, and it was not conducive to the packaging and stability of the prepared composite phase change material. Figure 4d is an internal sectional view of 8CP, from which the staggered distribution of its pore size can be seen more clearly, which has obvious advantages over CK and CH. Figure 4e shows the SEM diagram of 8CPK, and compared with Figure 4a, it can clearly be seen that there was a large amount of PEG on its internal and surface loads, indicating that its loading effect was good [27]. The SEM results demonstrate that PP showed an excellent activation effect during the carbonization process, promoted the formation of a multi-level pore structure frame, and obtained a carbon matrix with a high specific surface area, and PEG could be well-loaded.

**Figure 5:**
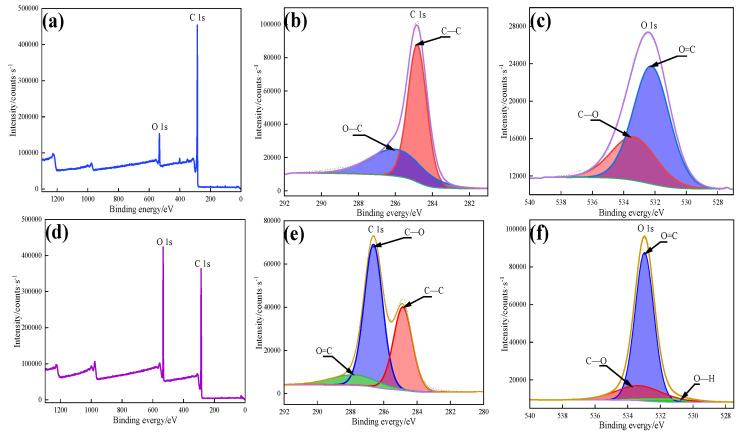
XPS spectrogram of (**a**,**d**) full spectra, (**b**,**e**) C1s spectra, (**c**,**f**) O1s spectra for 8CP and 8CPP, respectively.

To further determine the successful loading of PEG in the composites, XPS spectroscopy was performed, and the results are shown in Figure 5. From the XPS full spectrum of 8CP and 8CPP shown in Figure 5a,d, respectively, it can be seen that the peaks of C 1s and O 1s could be identified in the binding energy of 287 eV and 532.98 eV, respectively. From the C1s spectra of the 8CP and 8CPP materials, it can be seen that the presence of C elements in C–C, C=O, and C–O can be identified at the binding energy of 284.8 eV, 287.1 eV, and 286.4 eV, respectively, and the C–O and C=O of 8CPP were obviously stronger than the peaks of 8CP. From the O1s spectra of the 8CP and 8CPP materials, it can be found that the elements of O=C and O–C were identified in the binding energy of 532.98 eV and 533.48 eV, respectively, and the peak of 8CPP was obviously stronger than that of 8CPP, and the O peak in OH appeared at 530.98 eV of 8CPP. These results show that 8CP was loaded with a large amount of PEG, resulting in more C and O elements in 8CPP, and the appearance of OH further determined the existence of PEG; a similar view was also shown in this study of carbon-based phase change composites [28].

The specific surface area and microscopic properties of pores are important properties to measure the structure and loading causes of the prepared biochar matrix. Figure S1a shows the N_2_ absorbent and desorption isotherm of 8CP, which has a specific surface area of up to 1,082.2 m^2^/g calculated by the BET equation, which provides favorable conditions for loading a large number of phase change media. Similarly, Xie [29] reported the use of H_2_O_2_-modified coconut shell carbon as a support carrier with an increased specific surface area, allowing for the loading of more stearic acid. Li [30] found that fungal-derived carbon with a high specific surface area was loaded with more stearic acid compared to low specific surface area. The average pore size measured by the BJH method was 2.5378 nm. The results showed that the modified biochar had two pore size structures, microporous and mesoporous, which had an obvious lifting effect on the efficient adsorption of PEG [31]. The phase change of PEG in confined space can greatly reduce the leakage rate and reduce the subcooling degree. Combined with the SEM results, it can be seen that although SEM showed many porous structures of different sizes, there were relatively many micropores, which verifies the excellent packaging ability of the material for phase change media. Figure S1b shows the pore size distribution of 8CP. As shown in Figure S1b, the 8CP material had a wide pore size distribution and a large pore volume. Therefore, this porous structure constructed by PP activation is conducive to PEG loading to the carbon matrix.

**Figure 6:**
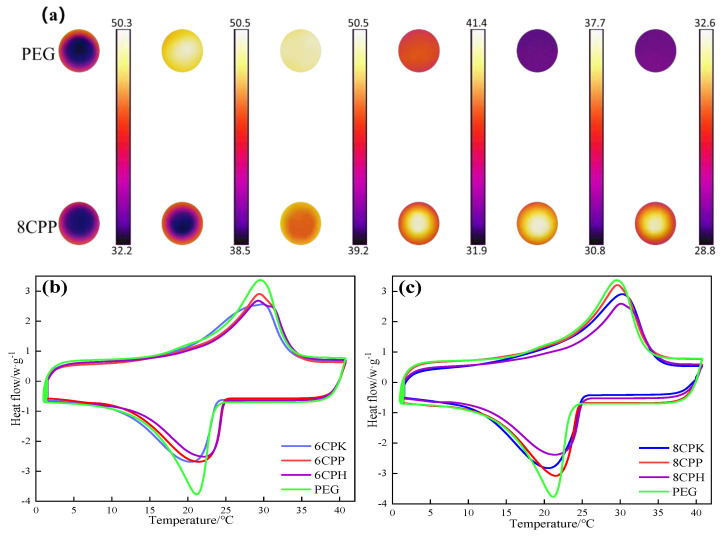
(**a**) Infrared thermal imaging of 8CPP and PEG; DSC of the composite material and PEG under activation at (**b**) 600 °C and (**c**) 800 °C.

The heat storage properties of the prepared composites were studied by DSC and infrared imaging technology. Infrared cameras were used to record the different temperature responses of the composite samples and PEG during heating and cooling. All samples containing a PEG mass of 5 g were compressed into wafer of the same size under equal pressure, then placed in a mold made of tin foil to avoid leakage and heated uniformly during the phase change. In addition, the temperature of the electric heating plate was kept at 50 degrees and the samples were cooled down at room temperature after reaching the maximum temperature. As shown in Figure 6a, the heating process of the two samples was sequential, resulting in the rapid cooling of PEG and 8CPP due to the influence of ambient temperature. From the comparison, it can be seen that PEG heated up faster during the heating process, and 8CPP had a significantly higher buffering capacity for heat than PEG. Moreover, the cooling rate of the composite materials was significantly slower than that of PEG. As described in the time–temperature curve in Figure S2, the temperature of PEG was always higher than 8CPP before 4000 s; between 4000 s and 5000 s, 8CPP gradually exceeded PEG and increased slightly; then, the temperature of PEG dropped to the initial temperature at 5670 s, and 8CPP was still close to 18 °C at this time. When PEG was the same as the ambient temperature, 8CPP still maintained a certain amount of heat, which fully reflects that 8CPP has good heat storage performance, indicating that the prepared composite can adjust the ambient temperature [32]. In Figure 6b,c, the pure PEG displayed a melting temperature (*T*_m_) of 17.32 °C, and the composite phase change material melting temperature had a range of 17.91 °C–21.19 °C, so it is believed that the rise in the phase-transition point in the composite can be considered as the confinement of PEG in the composites. While the crystallization temperature (*T*_c_) was 23.56 °C, all composite phase change materials had higher *T*_c_ than PEG, which reduced the phase change subcooling of the composite. The phase transition peak of melting and freezing in the composites was similar in shape to pure PEG, indicating that no chemical reaction occurred between PEG and the carbon carrier. The melting enthalpy of pure PEG (145.39 J/g, listed in Table 1) was higher than that of the three composites because the carbon carrier had no phase change heat generation. The composite phase change material with PP-modified biochar as the carrier had the highest phase change enthalpy at 600 °C and 800 °C for the carbon carriers prepared under the three modifications. In particular, the melting enthalpy of the PP-modified carbon composite phase change material at 800 °C reached 114.92 J/g. The high latent heat of phase change will have important applications in construction materials [33].

In order to evaluate the influence of support materials on the latent heat of phase change, we explored the intrinsic relationship between the structure and properties of the composite phase change materials. The cladding rate (*R*), impregnation rate (*E*), relative enthalpy efficiency (*λ*), and impregnation efficiency (*ψ*) were calculated according to Equations (1)–(4), to understand the relationship mechanism between the microstructure and the properties of the composite phase change materials.

(1) $$R=\frac{\Delta H_{m\text{-}\mathrm{PCM}}}{\Delta H_{m\text{-}\mathrm{PEG}}}*100\%$$

(2) $$E=\frac{\Delta H_{m\text{-}\mathrm{PCM}}+\Delta H_{c\text{-}\mathrm{PCM}}}{\Delta H_{m\text{-}\mathrm{PEG}}+\Delta H_{c\text{-}\mathrm{PEG}}}$$

(3) $$\lambda =\frac{\Delta H_{m\text{-}\mathrm{PCM}}}{\Delta H_{m\text{-}\mathrm{PEG}}*\omega }*100\%$$

(4) $$\psi =\frac{\frac{\Delta H_{m\text{-}\mathrm{PCM}}+\Delta H_{c\text{-}\mathrm{PCM}}}{R}}{\Delta H_{m\text{-}\mathrm{PEG}}+\Delta H_{c\text{-}\mathrm{PEG}}}$$

where *ω* reflects the mass fraction (80%) of PEG in the prepared phase change material.

**Figure 7:**
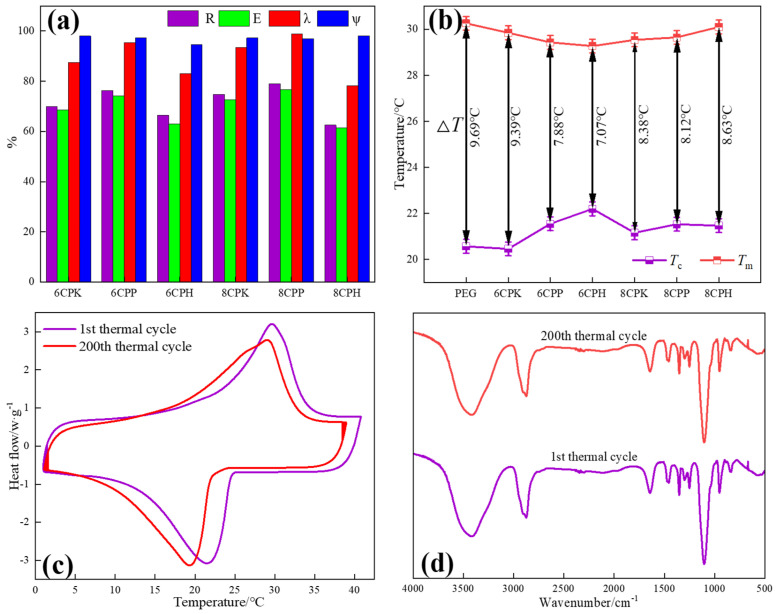
(**a**) *R*, *E*, *λ*, *ψ* of each composite material; (**b**) the subcooling degree of PEG and each composite material and PEG; (**c**) DSC diagram, and (**d**) FTIR diagram before and after 200 cycles of 8CPP.

Figure 7a shows the *R*, *E*, *λ*, *ψ* of each composite material. The terms *R* and *E* stand for the latent heat storage capacity of PEG in the composites and the effective impregnation of PEG in each modified biomass carbon porous structure, respectively [34]. The PEG crystallization in the composites was constrained by the molecular confinement, which makes it challenging for the PEG to conduct phase change interaction. In line with the findings of the DSC experiment, 6CPP and 8CPP had much higher *R* and *E* than the others, notably 8CPP. The latent heat loss for the composites decreases as the *λ* value increases because it measures how freely the PEG molecular chains may move. When 8CPP served as the carrier, the composite *λ* values were much greater, indicating that the porous structure of 8CPP facilitates PEG crystallization. *m* indicates the thermal storage capacity of the composites, and the *Ψ* of the composites in this study were all almost close to 100%, which indicates that each modified biomass carbon as a support material can effectively encapsulate PEG and effectively store and release heat through the phase change process. It can be seen from Table 2 that the *λ* and *Ψ* values in our work were higher than that in other studies, which indicates that the introduction of jujube carbon-based support materials is a suitable method to prepare novel PCMs with higher latent heat [35]. To further evaluate the heat storage performance of the composite, the subcooling degree and cycle stability were tested. It can be seen from Figure 7b that the phase change temperature of the composite material was lower than that of pure PEG, and the subcooling degree (Δ*T* = *T_mp_* − *T_cp_*, which is dependent on the melting and crystallization peak temperature [36]) of each composite material was also lower than that of PEG, indicating that the jujube charcoal carrier can reduce the subcooling degree of the phase change material to a certain extent. This is mainly due to the porous structure of carbon, with many fine pores distributed inside, which accelerates the crystallization rate of PEG and increases the crystallization temperature, thereby reducing the degree of supercooling. Among the various composite phase change materials prepared, the subcooling degree of 6CPH was the smallest, and the actual crystallization temperature was closer to the theoretical crystallization temperature. From the composite materials with three different activation methods, CPP showed that the effect of reducing the subcooling of PEG was more stable and excellent. As can be seen from the DSC curve in Figure 7c, the melting temperature and crystallization temperature of 8CPP were reduced after 200 thermal cycles. Observing the peak area, it can be seen that although the enthalpy value decreased, the amplitude was not very large, and Δ*H*_m_ and Δ*H*_c_ were only reduced by 4.3% and 4.1%, respectively, indicating that the prepared composite has good thermal cycling. It can be seen from the FTIR spectrum of Figure 7d that after 200 thermal cycles, the position and intensity of the characteristic absorption peak were almost unchanged, proving that after 200 cycles of PEG and carbon materials, no chemical reaction occurred, the chemical structure did not change, and its surface properties were almost unaffected by melting and crystallization. These results show that the carbon-based composite phase change materials prepared by PP activation have good thermal response, phase change reliability, and operational durability, and can be used for actual thermal energy storage and management. The thermal conductivity of composite materials is closely related to the application of the material [37]. It can also be seen from the thermal conductivity curves (Figure S3) that the thermal conductivity of pure PEG was about 0.3, and the thermal conductivity was poor, which needs to be further improved by the base material. The addition of the jujube charcoal base increased the thermal conductivity of the composite phase change material by about 0.1, which was not too high overall, so the material had good thermal insulation performance.

**Figure 8:**
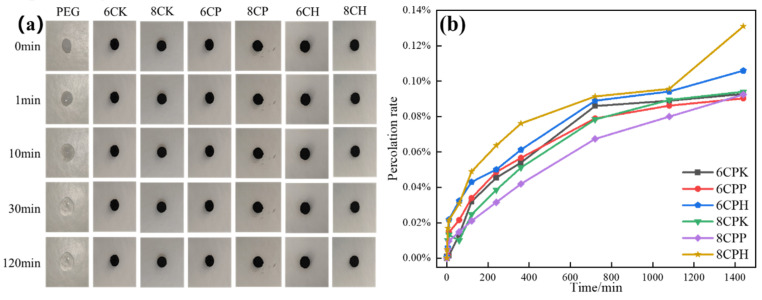
(**a**) Leakage of the composites and PEG at 30 °C; (**b**) leakage rate of the prepared composites.

The leakage of the composite material is directly related to the loading effect and packaging capacity of the material, and is related to the application of the material. In order to test the material leakage rate (the calculation is shown in Equation (5)), we pressed the PEG and composite phase change materials into discs of 0.6 mm in diameter and 0.2 mm in height on filter paper and heated them on a heating plate at 30 °C to observe the leakage of the materials. The composite materials were also placed on the heating plate for a long time, and the leakage rate was determined by weighing the mass of the materials after heating for a certain time. Figure 8a shows the leakage results of the composites. As can be seen from Figure 8a, the shape of PEG changed after 1 min at 30 °C and melted completely after 10 min, indicating that PEG is extremely unstable at 30 °C. For the prepared composite material, after 2 h, its corresponding mass was weighed, where there was almost no leakage, and the shape did not change. Figure 8 shows the leakage of the composites after 1400 min. We found that the leakage rate of all composites was less than 0.14% after a long time, and the leakage rate of the PP-treated carbon carrier composite phase change material was only 0.85%, which further demonstrates the excellent encapsulation ability and thermal stability of the carbon support for PEG [43].

(5) $$w=\frac{m_{0}-m_{1}}{m_{0}}$$

where *m*_0_ and *m*_1_ are the mass of the sample before heating and after heating for a certain time, respectively.

## 4. Conclusions

The biomass jujube charcoal-based composite phase change material was successfully prepared by the vacuum impregnation method using PP-activated jujube charcoal as the matrix and PEG as the phase change medium compared with the traditional acid and alkali activation. The prepared biomass jujube charcoal had a loose porous structure and staggered pore structure, which had a good encapsulation effect on PEG. Moreover, the positions of the characteristic peaks of the functional group and characteristic diffraction peaks of the composite material and PEG were similar, and no new obvious peaks existed before and after loading, indicating that the carbon material and PEG were physically combined. The PP activation process achieved satisfactory results. Compared with the traditional activation method, the specific surface area of CP was much larger than that of CK and CH, reaching 1082.2 m²/g, and had many staggered pore structures, which makes it the best loading effect on PEG, and its phase change enthalpy was also the highest. In addition, it had excellent performance in improving PEG subcooling and improved stability. In addition, the activation temperature also had an effect on the porous carbon structure, for example, 8CP had a better specific surface area and pore size than 6CP, making 8CPP obtain a higher enthalpy and thermal stability than 6CPP. The cycle stability of 8CPP was also very good, and the developed pore structure and surface functional groups ensured that the phase change enthalpy value only lost about 4% after 200 thermal cycles, which can achieve the purpose of green recycling. In a word, the prepared jujube charcoal composite phase change material has good thermal stability and encapsulation effect, possessing a superior porous carbon structure, especially after PP activation, which provides a reference value for the preparation of green recyclable biomass char composite phase change materials.

## Figures and Tables

**Table 1:** Phase transition temperature and phase change enthalpy of PEG and porous carbon materials.

Samples	Melting Temperature/°C	Enthalpy of Melt/J·g^−1^	Crystallization Temperature/°C	Enthalpy of Crystallization/J·g^−1^
PEG	17.32	145.39	23.56	143.3
6K	17.91	101.70	23.75	96.34
6P	21.19	110.96	24.65	103.26
6H	20.13	96.63	24.74	85.01
8K	19.21	108.63	24.95	101.03
8P	20.05	114.92	24.49	106.15
8H	20.80	90.97	25.04	86.25

**Table 2:** Comparison between the thermal energy storage characteristics of different PEG-based PCMs reported in the literature.

Samples	*R*/%	*E*/%	*λ*/%	*ψ*/%	Cited Literature
Potato char/PEG	77.20	73.30	90.44	94.94	[38]
Diatom char/PEG	64.94	61.53	98.10	94.75	[39]
Diatomite/PEG	60.83	54.85	86.9	90.17	[40]
Chitosan/PEG	72.95	70.26	91.2	96.31	[41]
Bone char/PEG	43.91	43.17	87.82	98.31	[42]
8CPP	79.04	76.58	98.80	96.88	This study

## Data Availability

Data are contained within the article.

## Supplementary Materials

The following are available online at https://www.mdpi.com/article/10.3390/nano13030552/s1, Figure S1. (a) The N2 adsorption and desorption curve of 8CP; (b) the pore size distribution of 8CP. Figure S2. Temperature rises curves of 8CPP and PEG. Figure S3. Thermal conductivity of composite phase change materials and PEG at 600 °C and 800 °C
