# Supplementary material for: Waste Plastic Polypropylene Activated Jujube Charcoal for Preparing High-Performance Phase Change Energy Storage Materials
Source: Nanomaterials (Basel). 2023 Jan 29;13(3):552. doi: 10.3390/nano13030552 (PMC9919484; doi:10.3390/nano13030552)
Supplement: Supplementary file 1 [file nanomaterials-13-00552-s001.zip › nanomaterials-2107866-supplementary.pdf]

Article

Waste Plastic Polypropylene Activated Jujube Charcoal for Preparing High-Performance Phase Change Energy Storage Materials

Xifeng Lv [1,2], Huan Cao [1], Rui Zhang [1], Xuehua Shen [1,*], Xiaodong Wang [2] and Fang Wang [3,*]

[1] College of Chemistry and Chemical Engineering, the National and Local Joint Engineering Laboratory of High Efficiency and Superior-Quality Cultivation and Fruit Deep Processing Technology of Characteristic Fruit Trees in South Xinjiang, Tarim University, Alar 843300, China
[2] State Key Laboratory of Organic-Inorganic Composites, Beijing 100029, China
[3] School of Environmental Engineering and Chemistry, Luoyang Institute of Science and Technology, Luoyang 471023, China.
* Correspondence: rainspirit_shen@163.com (X.S.); wangfang1116@163.com (F.W.).

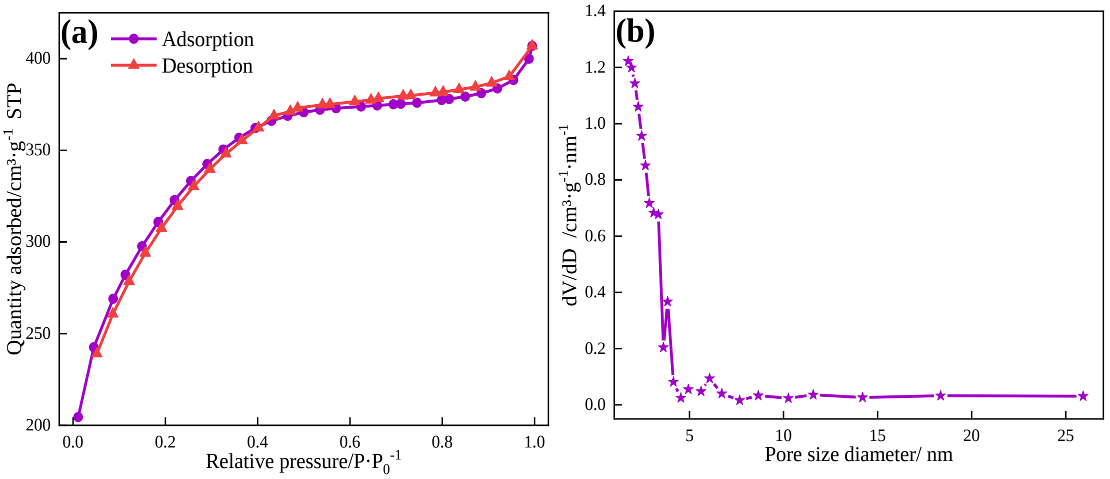

Figure S1. (a) The N_2 adsorption and desorption curve of 8CP; (b) the pore size distribution of 8CP.

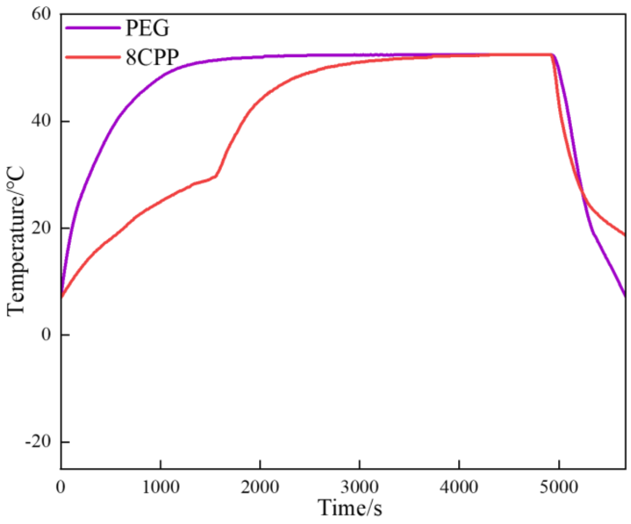

Figure S2. Temperature rises curves of 8CPP and PEG.

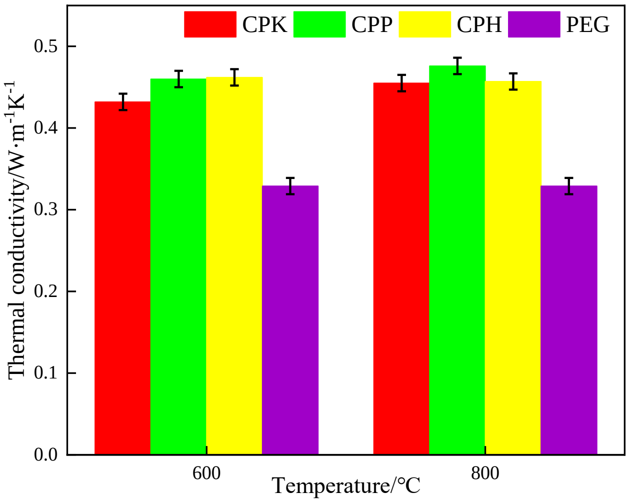

Figure S3. Thermal conductivity of composite phase change materials and PEG at 600 °C and 800 °C
